# Supplementary material for: Identification of photoperiod-induced specific miRNAs in the adrenal glands of Sunite sheep (Ovis aries)
Source: Front Vet Sci. 2022 Jul 22;9:888207. doi: 10.3389/fvets.2022.888207 (PMC9354845; doi:10.3389/fvets.2022.888207)
Supplement: Supplementary file 1 [file Data_Sheet_1.ZIP › Supplementary file/miRDeep2 file.zip!/miRDeep2 file/Novel_Structure/SPLP42A3/Novel_85.pdf]

Provisional ID	:	NC_019467.2_19304
Score total	:	2.4
Score for star read(s)	:	-1.3
Score for read counts	:	0
Score for mfe	:	2.1
Score for randfold	:	1.6
Score for cons. seed	:	
Total read count	:	55
Mature read count	:	55
Loop read count	:	0
Star read count	:	0

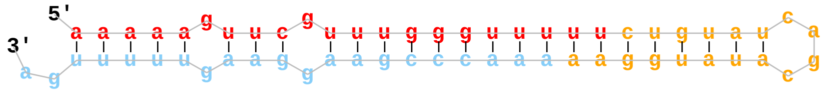

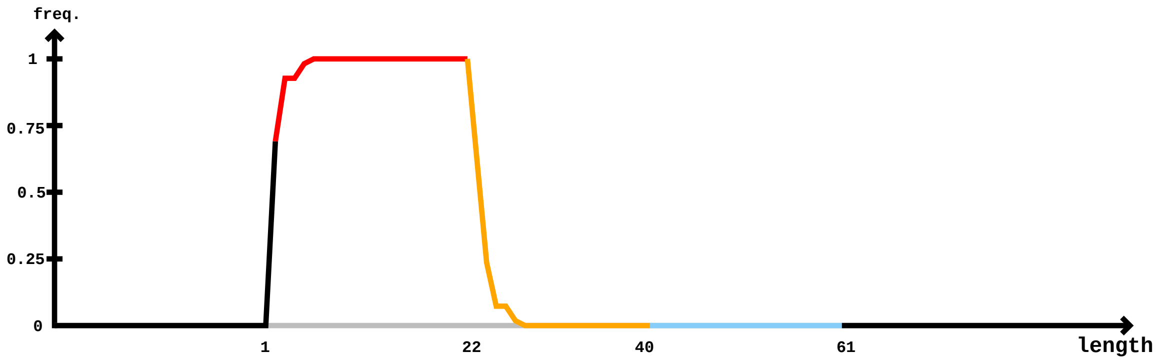

Mature **Star**

```
5'- gcaucauauauuggguuggcgaaaaaguucguuuggguuuuucuguaucagcauauggaaaaacccgaaggaaguuuuugaacaacccaauaugauauguucaaaaugga  -3'    exp
    ((((..((((((((((((...(((((.(((.((((((((((((((((....)))))))))))))))).))).)))))...))))))))))))..)))).........   reads    mm    sample
    .....................aaaaaguucguuuggguuuuG....................................................................   1        1     seq
    .....................aaaaaguucguuuggguuuuu....................................................................   24       0     seq
    .....................aaaaaguucguuCggguuuuu....................................................................   5        1     seq
    .....................aaaaaguuUguuuggguuuuu....................................................................   1        1     seq
    .....................aaaaaguucguuuggguuuuC....................................................................   2        1     seq
    .....................aaaaaguuUguuuggguuuuuc...................................................................   2        1     seq
    .....................aaaaaguucguuuggguuuuuA...................................................................   1        1     seq
    .....................aaaaaguucguuuggguuCuuc...................................................................   1        1     seq
    .....................aaaaaguuUguuuggguuuuucu..................................................................   1        1     seq
    ......................aaaaguucguuuggguuuuuA...................................................................   5        1     seq
    ......................aaaaguucguuCggguuuuucu..................................................................   2        1     seq
    ......................aaaaguucguuuggguuuuucu..................................................................   5        0     seq
    ......................aaGaguucguuuggguuuuucu..................................................................   1        1     seq
    ........................aaguucguuuggguuuuCcugu................................................................   2        1     seq
    ........................aaguucguuuggguuuuCcugua...............................................................   1        1     seq
    .........................aguucguuuggguuuuCcugu................................................................   1        1     seq
```
